# Assessment of food habits in children aged 6–12 years and the risk of caries

**DOI:** 10.1080/13102818.2014.989180

**Published:** 2014-12-12

**Authors:** Liliya Doichinova, Peter Bakardjiev, Milena Peneva

**Affiliations:** ^a^ Department of Pediatric Dentistry, Faculty of Dental Medicine, Medical University of Sofia, Sofia, Bulgaria

**Keywords:** food habits, children, caries risk, carbohydrate intake, dental caries

## Abstract

Food is necessary for the proper growth and development of children. The excessive intake of low-molecular carbohydrates constitutes a serious health issue, which has an unfavourable impact on the dental health status. The aim of this study was to assess the food habits in healthy children aged 6–12 years and the effect on their oral risk profile. The study included 100 children. The assessment of their nutrition was done with the help of a seven-day reproduction of the food intake and a survey used to determine their underlying food habits and preferences. The results revealed unbalanced nutrition of the children and increased intake of simple sugar, which will increase the risk of development of dental caries. The observed high levels of DMFT (number of decayed, missing and filled teeth) in 54% of the children is a logical result of the frequent intake of sugary foods and beverages for a long period of time, as this will increase the acid production by microorganisms in dental plaque, which is one of the leading etiologic factors for the development of caries. It is necessary for dentists to administer control over the carbohydrate intake and the food habits of children, as well as to encourage non-cariogenic diet in order to keep their good oral health.

## Introduction

Food is necessary for the proper growth and development of children. It is important for the support of oral and physical health, the enhancement of the powers of resistance and continued renewal of the substances in the cells and tissues in children.

According to the World Health Organization, the diet has an important role in the prevention of oral diseases, including dental caries, dental erosion, defects in development, diseases of the oral mucosa and periodontal diseases.[1] Dental caries eventually leads to tooth loss, which in turn impairs the chewing ability, causing avoidance of hard and fibrous foods, including fruits, vegetables and whole grains.[1] An effective means of caries prevention is consumption of fluoridated water coupled with reduction in the intake of non-milk extrinsic sugar.[2]

The excessive intake of low-molecular carbohydrates constitutes a serious health issue, which has an unfavourable impact on the dental health status.[3]

The aim of this study was to assess the food habits in healthy children aged 6–12 years and the effect on their oral risk profile.

## Subjects and methods

The study covered 100 children aged 6–12 years. The assessment of their nutrition was done with the help of a seven-day reproduction of the food intake and a survey used to determine their underlying food habits and preferences. In order to obtain a relevant idea of the regular food diet, the child or the parent was asked to document all nutrition intake (food and drinks) for a period of seven days, in a diary. The week or the seven-day span was chosen so as to be a period corresponding with the child's normal everyday life.

The intensity of the dental caries in children was assessed on the basis of the Klein and Palmer (1938) decay–missing–filled (DMF) index.[4] Depending on the results, a child can fall within one of the three risk groups: low risk, up to 2 DMFT (number of decayed, missing and filled teeth); medium risk, up to 4 DMFT and high risk, above 4 DMFT. Relationships between perceptions of healthiness of eating patterns were analysed using chi-squared statistics, χ^2^ test.

The study was approved by the Medical Ethics Board at the Medical University of Sofia. All participants were informed about the purpose of the study and gave their written informed consent.

## Results and discussion

**Figure 1.**
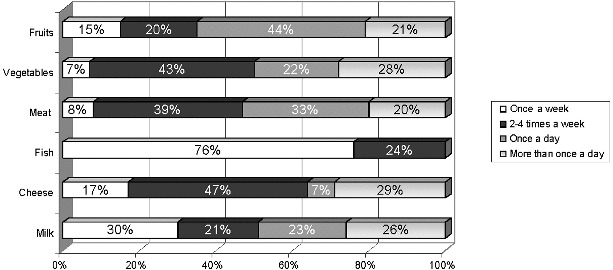
Food preferences in children aged 6–12 years.

The analysis of the one-week food diet regime is shown in Figure 1 and it documents an unbalanced diet.

The results are a cause for concern, as milk and dairy products were rarely present in the diet of more than half of the children. The majority of them were reported to eat fish only once a week. Increased consumption of meat and meat products was observed in half of the children.

Healthy eating for children and students is a priority of the Ministry of Health of the Republic of Bulgaria (Ordinance No 37, 2009).[5] This is achieved by providing a complete and varied diet, daily consumption of fruits and vegetables, adequate intake of milk and milk products and foods rich in protein, increased consumption of whole grains.

The Ordinance gives the necessary amounts of food for protection of oral health. An assessment of the dietary intake of these foods by the children who took part in our study is shown in Table 1. The data indicate that the quantity of the basic products consumed was not sufficient for protection from dental caries.

**Table 1.** Daily intake of protective foods for oral health for students 6–12 years of age.

	Age (years)			
	6–10		10–12	
Products (net)	Recommended quantity (g)	Consumed quantity (g)	Recommended quantity (g)	Consumed quantity (g)
Vegetables (net)	300/330	210	300/330	190
Fruit (net)	220/250	160	300/350	210
Milk (net)	400	305.7	400	298.3
Cheese (net)	30	23.2	40	20.4
Meat, meat products total (net)	55	41.5	80	62.5
Fish (net)	30	20.1	40	29.3

The results showed that children consume very low quantities of milk and milk products. The levels consumed are totally insufficient for the mineralization of the teeth that have not yet emerged and insufficient to provide local protection of the ones emerged. Consumption of dairy products has a positive impact on food diets of children, especially in school.[6]

Milk and milk products are a group of foods that must be a priority in child feeding. They are rich in nutritious protein, group A and B vitamins and easily assimilated calcium, crucial to dental health.[6]

Poor oral hygiene, lack of parental control and appropriate health knowledge, together with frequent consumption of cariogenic foods, in addition to social demographic characteristics are the main risk factors for the development of caries in surveyed schoolchildren.[7,11] A similar case was observed regarding meat and fish. Their consumption quantities were also shown to be insufficient.

The quantities of the consumed fruits and vegetables were also less than the recommended ones. This is a disturbing fact, as this food group in the food pyramid is rich in all group B vitamins and all water-soluble vitamins, including vitamin C. Vegetables are richer in water-soluble vitamins. Fruits are indispensable food, rich in vitamins, minerals and organic acids.[6,7]

**Figure 2.**
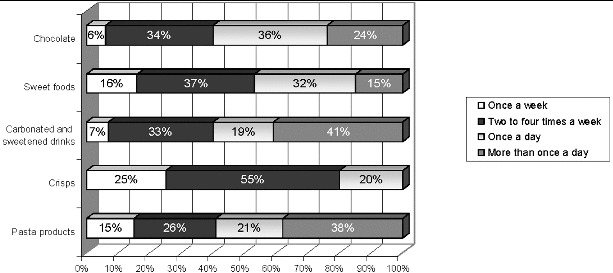
Weekly intake of carbohydrate foods in children aged 6–12 years.

For a clearer picture of carbohydrate intake as one of the most prominent risk factors for the development of dental caries, the weekly carbohydrate intake in children was analysed (Figure 2).

In the everyday diet of most children there were many risk foods of the ‘junk food’ type: chocolate, sweets, snickers, potato crisps, corn sticks. Such foods are appealing and have a pleasant taste (mostly sweet); they are packed in colourful shiny packages in the form of sticks, chocolate bars or sweet drinks in boxes. Their high sugar content causes a major concern as it tremendously raises the risk of caries. Other foods such as corn sticks, corn snacks, popcorn, crisps, French fries are very tasty and have an appealing aroma and colour.

A worrying trend of frequent consumption of non-alcoholic fizzy drinks was found in 2/3 of the children. Due to the high sugar content, this creates a high risk of caries development. Energy drinks are a favourite amongst some children, who consume them regularly.

Dental caries is a common, chronic disease of childhood.[1,6] What is most important for its prevention is controlled sugar intake. It has been shown that when the intake of free sugars is less than 15 kg per person per year, the level of dental caries is low.[1] Global recommendations encourage diets with a high content of fruits, vegetables and a low content of added sugars and fats for the protection of oral and general health.[1]

The risk of caries is highest if sugar confectionery is consumed most frequently and is in a form that is retained in the mouth for a longer period of time. Guidelines in training parents and advising children include diets that avoid frequent consumption of juice or other sugar-containing products that cause tooth decay, and promote healthy eating habits consistent with the Food Guide Pyramid.[8–10]

Food consumption affects the state of the teeth, the most significant effect being the local effect of food containing free sugars on the tooth surface and development of caries and erosion. The frequency of intake of such foods should be limited to a maximum of four times a day. It is the responsibility of the national authorities in each country.[8,11–14]

Table 2 shows the caries development risk factor of low carbohydrate food intake in children. The participants could be separated into three risk groups according to the data gathered.

**Table 2.** Carbohydrate intake and intensity of dental caries in children aged 6–12 years.

Risk factor carbohydrate intake	Low risk (limited intake)	Medium risk (seldom between meals)	High risk (frequent intake)
Children (*n* = 100)	20 (20%)	26 (26%)	54 (54%)
DMF(T+t)	1.8	3.1	5.2

Note: Seldom between meals/frequent intake χ^2^ = 16.3 (*p* < 0.001).

Limited intake/frequent intake χ^2^ = 24.8 (*p* < 0.001).

Limited intake/seldom between meals χ^2^ = 0.01 (*p* > 0.05).

In terms of food regime, only 26% of the children were reported to have a low intake of carbohydrates during proper meals. Only 20% of the children fell in the group that rarely consume simple sugars in between meals, whereas in most children (54%) the intake of low-molecular carbohydrates can be considered a serious risk factor for the development of caries. The correlation between carbohydrate intake of simple sugars and the intensity of dental caries in the children surveyed is also presented.

The high values of the DMFT index (above 4) that we registered in 54% of the children are a logical result based on the reported frequent consumption of sugary foods and drinks, which can, for a prolonged period of time, increase the production of acid by microorganisms located in the dental plaque, one of the leading etiological factors for the development of caries. The results showed a correlation between the frequency of low-density carbohydrates intake and the intensity of dental caries within the studied cases. Childhood is a key period for acquiring proper food habits. During puberty these habits are firmly established and in many children remain the same throughout the rest of their lives.

Parents have a very important role in training and educating children in healthy eating habits. The study of Rajab et al. [16] shows that 80% of the parents knew about the harmful effect of sugar and 79% thought that poor oral hygiene may induce dental caries. In addition to proper oral hygiene (79%) and restriction of sugar/sweets (42%), 36% of the parents stressed the importance of regular dental visits for the prevention of dental disease in children. This indicates the need for health education, since there is a gap between dental knowledge and attitudes of parents and dental practices in healthcare. School-based programmes to promote oral health can be expected to affect the behaviour of children and their parents in order to avoid further deterioration of oral health.[14–16] A questionnaire completed by parents is commonly used to describe the frequency of meals per day. The data collected can be analysed to assess how often the children consume foods listed as harmful to their dental health. The number of caries in each child is recorded after that.[18,19] Programmes for health promotion and health care providers should target patients and caregivers in order to reduce the risk of caries associated with the consumption of soft drinks.[6] Children who consume more soft drinks than milk and 100% fruit juice, are with a higher risk of dental caries.[20]

The relationship between the number of caries has been analysed as the sum of DFS and DMFS indices and consumption of cariogenic foods in a population of children aged between 6 and 10, which are with predominantly low risk of caries.[1]

There are reports that show no connection between the number of daily meals and the increase of caries frequency. However, there is correlation between proximal caries development and sweets consumption on a daily basis. For example, children categorized with high caries risk have an increased sweets intake compared to children with low caries risk.[11]

In this context, it is of particular concern that food commercials targeting children generally reflect a diet that is associated with increased risk of obesity and dental caries in childhood.[7] Consultations regarding food diet are one of the key factors in the prevention of dental caries and reducing the risk of its development. The specific recommendations for each child should be given after diagnosing any food deficiencies and/or excesses in their regular food regime, and alternatives should be provided. Complex actions such as the European Commission ‘School Fruit’ Scheme [21] and the amendments for offering healthy foods in schools and kindergartens will in time create a healthy food model for children. The frequent consumption of low-molecular carbohydrate foods such as pasta products, sweet foods and fizzy drinks with added sugar will, in more than half of the children studied, increase the risk of caries development,[1,3,12] as most children do not have a proper food regime.

The dentist must regulate the carbohydrate intake and food habits in children and encourage a healthy diet that helps the prevention of caries and helps maintain an overall good oral health.

Rashkova et al. determine oral hygiene, carbohydrate meal viscosity of saliva and its buffer capacity, the incidence of caries of their parents and the social status of the family as the highest risk factors for the development of caries in children studied in Bulgaria. For the management of dental caries is required an integrated approach, including a risk assessment of dental caries and correction of risk factors and recommendations for healthy behaviours, especially in terms of diet and oral hygiene by dentists.[22]

## Conclusions

Our survey on the eating habits and caries risk in 100 children from Bulgaria aged 6–12 years showed that there is low consumption of caries protective foods at the expense of food bearing a high risk of caries. The results highlight the important role of the dentist in giving consultations regarding food diet for the prevention of dental caries and reducing the risk of its development. The specific recommendations for each child should be given after diagnosing any food deficiencies and/or excesses in their regular food regime, and alternatives should be provided. Complex actions such as the European Programme ‘School Fruit’ and the amendments for offering healthy foods in schools and kindergartens should be encouraged.
